# Protein expression pattern of the molecular chaperone Mdg1/ERdj4 during embryonic development

**DOI:** 10.1007/s00418-020-01881-x

**Published:** 2020-05-07

**Authors:** Lea Daverkausen-Fischer, Myriam Motyl-Eisemann, Margarethe Draga, Martin Scaal, Felicitas Pröls

**Affiliations:** 1 Institute of Anatomy II, University of Cologne, Faculty of Medicine, Joseph-Stelzmann Str. 9, 50931 Cologne, Germany; 2 Klinikum Leverkusen Klinik für Kinder- und Jugendliche, 51375 Leverkusen, Germany

**Keywords:** Molecular chaperone, Hsp40 protein, Mdg1/ERdj4/mDjB9, Chick, Mesenchymal–epithelial transition (MET), Epithelial–mesenchymal transition (EMT)

## Abstract

The vertebrate-specific co-chaperone Mdg1/ERdj4, which is localized in the endoplasmic reticulum, controls the folding and degradation of proteins. We characterized its protein pattern during chick embryonic development. During early development, Mdg1/ERdj4 protein is present in mesenchymal and epithelial cells. In mesenchymal cells, it has a salt and pepper pattern. In contrast, during epithelial tissue differentiation, Mdg1/ERdj4 marks the basal and/or apical compartment of epithelial linings. The distinct protein pattern in epithelial tissue might point to its role in organizing and maintaining the epithelial structure. This could be achieved, e.g. by controlling folding and secretion of membrane-bound receptors or by inhibiting the IRE1α–Xbp1s–SNAI1/2-induced mesenchymalization. High Mdg1/ERdj4 protein levels are maintained in tissue with sustained secretory activity as in ependymal cells or enterocytes, substantiating its important role for secretion. We conclude that the transient elevation of Mdg1/ERdj4 protein levels controls the differentiation of epithelial linings while constitutive high levels are closely linked to secretory activity.

**Electronic supplementary material:**

The online version of this article (10.1007/s00418-020-01881-x) contains supplementary material, which is available to authorized users.

## Introduction

Due to their induction in heat-shocked cells, chaperones have originally been described as heat shock proteins (Hsps). We now know that only a subset of chaperones is induced in heat-shocked cells. Chaperones are essential for proper folding of proteins and for degradation of irreversibly denatured proteins. They constitute the protein quality control system in every single cell and are essential for maintenance of the cellular ionic and protein homeostasis. Since the on and offset of protein translation and protein transport processes constantly challenge cellular homeostasis, chaperones are also required for a variety of normal cellular processes such as proliferation, cell differentiation and migration. The number of chaperones largely varies in different species. In humans, 13 members of the Hsp70 family and 49 different co-chaperone proteins have been identified until today. It is largely unclear why so many members of the chaperone families exist. Yet, some of them are localized in distinct subcellular compartments, others exhibit tissue- or developmental-specific expression (Vos et al. 2008). The onset of developmentally expressed chaperones starts very early in embryogenesis. The cytosolic, non-inducible Hsc70 for example, is expressed during early gastrulation at the border of the primitive groove and during neurulation in the neural fold and tube, the sinus rhomboidalis and the primitive streak (Vega-Nunez et al. 1999). The cytosolic, heat shock inducible Hsp70, is constitutively expressed during oogenesis (Angelier et al. 1996), but also specifically during maturation in erythroid cells (Banerji et al. 1984, 1987; Ribeil et al. 2007). Knockout mice revealed that the ER-specific Hsp70 protein BiP/GRP78 is essential for embryonic survival at periimplantation stage. BiP knockout mice die due to increased apoptosis of the inner cell mass and the subsequent inability to hatch from the zona pellucida (Luo et al. 2006).

The folding capacity of Hsp70 proteins is facilitated by members of the Hsp40 co-chaperone family. Hsp40 proteins, also named DnaJ-like proteins, are characterized by the evolutionary strongly conserved J-domain, which catalyses the ATPase activity of Hsp70. Their target specificity is determined by the non-J-domain containing region (Hennessy et al. 2005). Tissue specificity as well as fundamental functions have been attributed to a variety of Hsp40 co-chaperones (Berruti et al. 1998; Hunter et al. 1999; Fernandez-Chacon et al. 2004; Lo et al. 2004; Gotz et al. 2005; Terada et al. 2005).

Mdg1, also known as ERdj4, Dnajb9, or mDj7, is a member of the Hsp40 co-chaperone family. It is solely expressed in vertebrates and has first been described in 2001 due to its transient upregulation in differentiating microvascular endothelial cells (Prols et al. 2001). Its J-domain is promiscuous: it can stimulate the ATPase activity of Hsp70 and of the ER-specific Hsp70 protein BiP (Prols et al. 2001; Shen et al. 2002). In non-stressed cells, Mdg1/ERdj4 is predominantly located in the ER compartment, where it co-chaperones BiP (Prols et al. 2001; Shen et al. 2002; Berger et al. 2003; Kurisu et al. 2003). It can be hypothesized that within the ER compartment Mdg1/ERdj4 is necessary for folding, maturation and secretion of specific target protein(s). In accordance with this are the high expression levels of Mdg1/ERdj4 in secretorily active tissue (Prols et al. 2001; Shen et al. 2002). Furthermore it has been demonstrated that Mdg1/ERdj4 plays a fundamental role in the degradation of target proteins via the ER-associated degradation pathway (Dong et al. 2008; Buck et al. 2010). Mdg1/ERdj4 was shown to promote degradation of mutant surfactant protein C, wildtype and mutant insulin as well as of the epithelial sodium channel (Dong et al. 2008; Buck et al. 2010). Recently, Mdg1/ERdj4 has been described to prevent maturation of aggregated amyloid beta peptides, which are associated with Alzheimer’s disease (Hoshino et al. 2007). Besides this, high Mdg1/ERdj4 protein levels have been shown to lower the metastatic potential of tumour cells (Isachenko et al. 2006).

We investigated the localization of Mdg1/ERdj4 during embryonic development by immunohistochemistry of chick embryos at various developmental stages. In this study, we show that Mdg1/ERdj4 protein is predominantly expressed in the basal and/or apical compartment of epithelial linings. The characteristic pattern is lost during maturation of the tissue. Accordingly, we assume that, during embryonic development, Mdg1/ERdj4 is mainly involved in organization of epithelial borders while its presence in secretorily active tissue points to its role in secretion processes.

## Material and methods

### Generation of the Mdg1/ERdj4 antibody

Rabbit polyclonal anti-Mdg1/ERdj4 antibody was generated by immunization of rabbits with synthetic oligopeptide H2N-CQNQNTRSKKHFENH-CONH2 (corresponding to Mdg1/ERdj4 aa 127–140). Synthesis of the peptide and the immunization procedure were performed by Eurogentec (Belgium). Specificity of the antibody was verified by Western blot analyses and immunohistochemistry (see Supplementary Fig. 1).

### Immunohistochemical staining

Fertilized eggs were incubated at 37 °C (80% humidity) for the times indicated and staged according to Hamburger and Hamilton (Hamburger and Hamilton 1951). Chick embryos of various developmental stages were fixed 2 days in paraformaldehyde (4%), transferred to and embedded in paraffin and sectioned. Organs were isolated from chick embryos of various stages, fixed for 2 days in Serra (60% ethanol, 30% formaldehyde, 10% acetic acid), embedded in paraffin and sectioned. Sections were deparaffinized in xylol and rehydrated in ethanol. Blocking of endogenous peroxidase activity was performed by incubating the slices in hydrogen peroxide for 20 min. Sections were microwaved for 10 min in citrate buffer (pH 6.0) for antigen retrieval and blocked with avidin/biotin (Biomeda) and normal goat serum (Perbo Pierce Laboratories). Slides were incubated with rabbit anti-Mdg1/ERdj4 peptide antibody (1:1000) over night at 4 °C followed by incubation with Multilink (Biogenex) (diluted biotinylated anti-immunoglobulin with carrier protein) for 20 min. Signal amplification was performed using streptavidin conjugated with alkaline phosphatase (Biogenex, Multilink antibody). Staining was performed using Fuchsin Substrate Chromogen System (DAKO) according to the manufacturer’s instructions. Nuclei were counterstained with hematoxylin (Merck). Negative controls (without incubating the sections with primary antibody) were routinely performed in parallel (see Supplementary Fig. 1). Immunohistochemical stainings were documented using a Zeiss Axioscope 5 microscope equipped with Zeiss AxiocamMR camera with a resolution of 1300 × 1030 and 12-bit digitization.

## Results

Immunohistochemical stainings were performed to visualize the localization of Mdg1/ERdj4 protein during embryonic development.

### Mdg1/ERdj4 protein pattern in the paraxial and lateral plate mesoderm

**Fig. 1:**
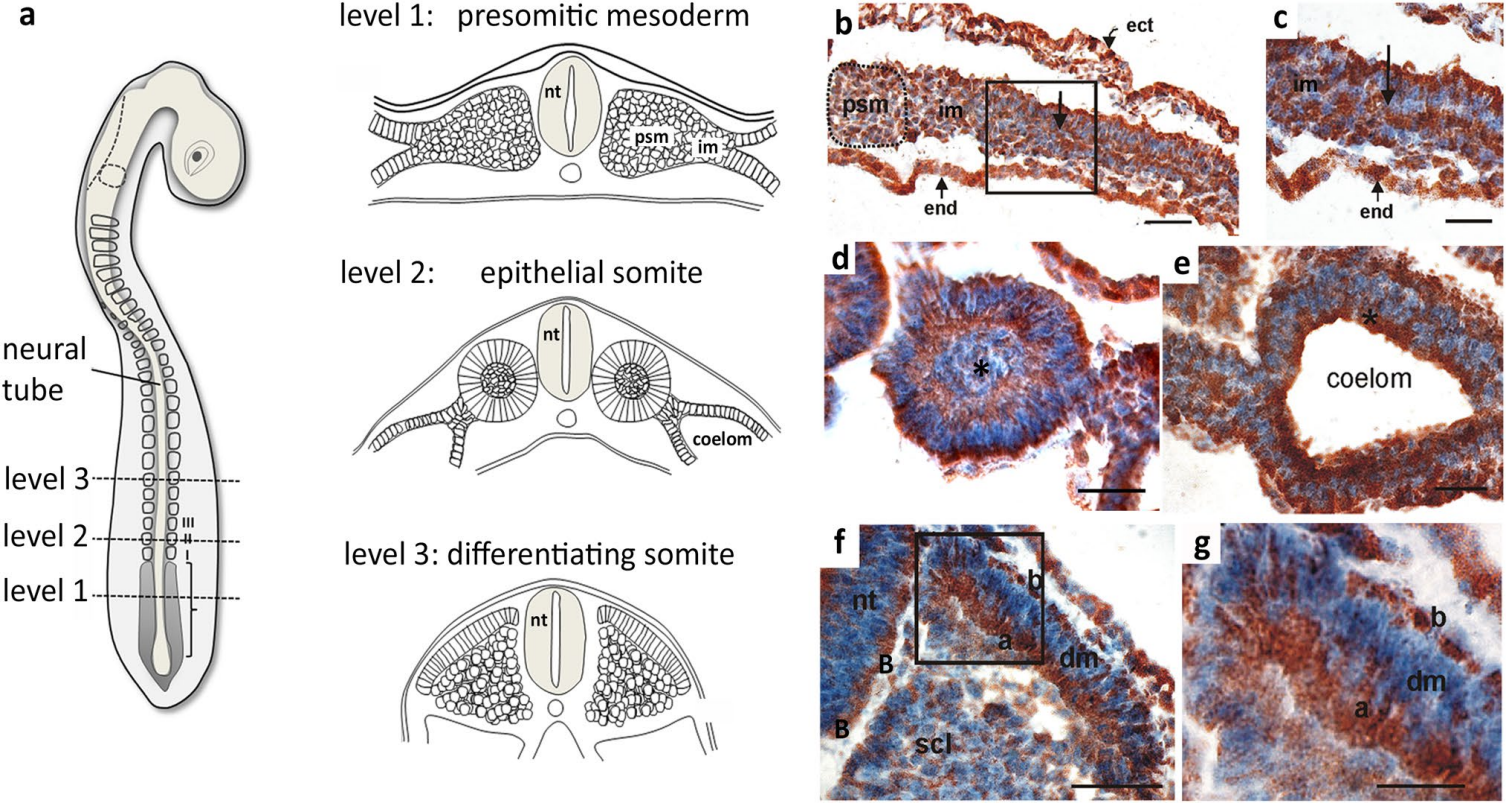
Mdg1/ERdj4 protein pattern in the paraxial mesoderm. All sections were counterstained with hematoxylin to visualize cellular nuclei. **a** Schematic illustration of a chick embryo at day 2, developmental stage HH12 (on the left: dorsal view). On the right: schematic illustration of coronal sections at three different levels. At level 1, cells in the presomitic mesoderm (psm) and the intermediate mesoderm (im) are mesenchymal. MET is initiated in the lateral plate mesoderm where cells become epithelial. At level 2, the somites are epithelial balls with a central mesenchymal somitocoel, the lateral plate mesoderm is epithelialized and delimits the coelomic cavity. At level 3, the epithelial somites are differentiated into the epithelial dorsolateral dermomyotome and the mesenchymal ventromedial sclerotome. nt (neural tube). **b** HH-stage 12, coronal section at level 1 of the presomitic mesoderm. All cells in the section are Mdg1/ERdj4 positive. The distribution of Mdg1/ERdj4 protein polarizes in cells that form an epithelium (rectangle). The arrow in the rectangle indicates the mesenchymal–epithelial transition zone (MET) at which the polarized Mdg1/ERdj4 distribution initiates. No polarized Mdg1/ERdj4 distribution is observed in the epithelial ectoderm (ect) or endoderm (end). Scale bar 50 µm. **c** Magnification of the MET zone marked in **b**. Scale bar 30 µm. **d** HH-stage 12, coronal section at level 2 of the epithelial somite II. Polarized Mdg1/ERdj4 protein pattern with highest ERdj4/Mdg1 amounts at the basal border of the epithelium. At the apical site that faces the mesenchymal somitocoel (*), the Mdg1/ERdj4 protein is not as tightly packed as at the basal site. Scale bar 30 µm. **e** HH-stage 12, coronal section at level 2 of the epithelial somite II. High amounts of Mdg1/ERdj4 protein accumulate at the apical and basal borders of the somato- and splanchnopleura. The apical border of the somatopleura that faces the coelomic cavity is marked (*). Scale bar 30 µm. **f** HH-stage 13, Coronal section at level 3 showing a somite that differentiated into epithelial dermomyotome (dm) and mesenchymal sclerotome (scl). The Mdg1/ERdj4 protein distribution is polarized in the epithelial dermomyotome with predominant accumulation at the apical (a) basal and (b) border. Note also the polarized apical and basal Mdg1/ERdj4 distribution in the epithelial neural tube (nt). Scale bar 50 µm. **g** Magnification of the rectangular zone marked in **h** showing the dorsomedial lip of the dermomyotome with Mdg1-/ERdj4-positive basal (b) and apical (a) borders. Scale bar 20 µm

During early embryonic development, the mesoderm is structured to form the axial, paraxial and lateral plate mesoderm. The axial mesoderm consists of the notochord. Its major function is the secretion of factors that induce the differentiation of surrounding tissue, as e.g. in the overlying ectoderm it induces the neuroectoderm, which gives rise to the entire central nervous system. The paraxial mesoderm, which is located at either side of the neural tube, gives rise to the vertebral column, dermis and musculature (Scaal 2016). The primary structures of the paraxial mesoderm are the metamerically organized somites. The paraxial mesoderm is of particular interest when investigating transformation processes, in which mesenchymal cells become epithelial (MET, mesenchymal–epithelial transition) or vice versa, when epithelial cells become mesenchymal (EMT, epithelial–mesenchymal transition). While the presomitic mesoderm is mesenchymal, epithelialization is initiated at its cranial zone where the somites are preformed. The somites that bud off are ball shaped with epithelial cells at the outer side that enclose mesenchymal cells, the so-called somitocoel. Maturation of these epithelial ball-like somites gives rise to the dorsolateral dermomyotome and the ventromedial sclerotome. While the dermomyotome maintains the epithelial structure, somitic epithelial cells located ventromedially become mesenchymal to form the sclerotome (see the schematic illustration of maturation of the paraxial mesoderm Fig. 1a). The primarily mesenchymal lateral plate mesoderm gives rise to the epithelial somato- and splanchnopleura, which enclose the coelomic cavity, the major cavity of the trunk, in which all intraperitoneal organs become surrounded by the splanchnopleural-derived peritoneum.

Immunohistochemical stainings of chick embryos at day 2 (HH-stage 12 and 13) revealed that the co-chaperone protein Mdg1/ERdj4 is expressed in all cells of the paraxial and lateral plate mesoderm. Very interestingly, the even protein distribution in the mesenchymal cells becomes reorganized during MET, a process first evidenced during epithelialization of the lateral plate mesoderm (Fig. 1b, c). MET is also a central process during somitogenesis, i.e. when the epithelial somites are formed from the presomitic mesoderm. Again the localization of the Mdg1/ERdj4 protein changes with highest amounts at the apical and basal zones of the epithelial layer while the central somitocoel maintains the mesenchymal-specific salt and pepper protein pattern (Fig. 1d). The epithelial sheets of the lateral plate mesoderm, termed somato- and splanchnopleura, surround the coelom and maintain the epithelial-specific Mdg1/ERdj4 protein pattern with high levels at the apical and basal regions (Fig. 1e). During further maturation of the somites, the dorsolateral dermomyotome, which remains epithelial maintains the epithelial-specific protein pattern with highest Mdg1/ERdj4 amounts at the apical zone. In contrast, the sclerotomal cells, which had undergone mesenchymalization (EMT) regain the mesenchymal-specific protein pattern with Mdg1/ERdj4 protein being primarily located in the perinuclear endoplasmic reticulum (Fig. 1f, g).

### Mdg1/ERdj4 protein pattern in the central nervous system

**Fig. 2:**
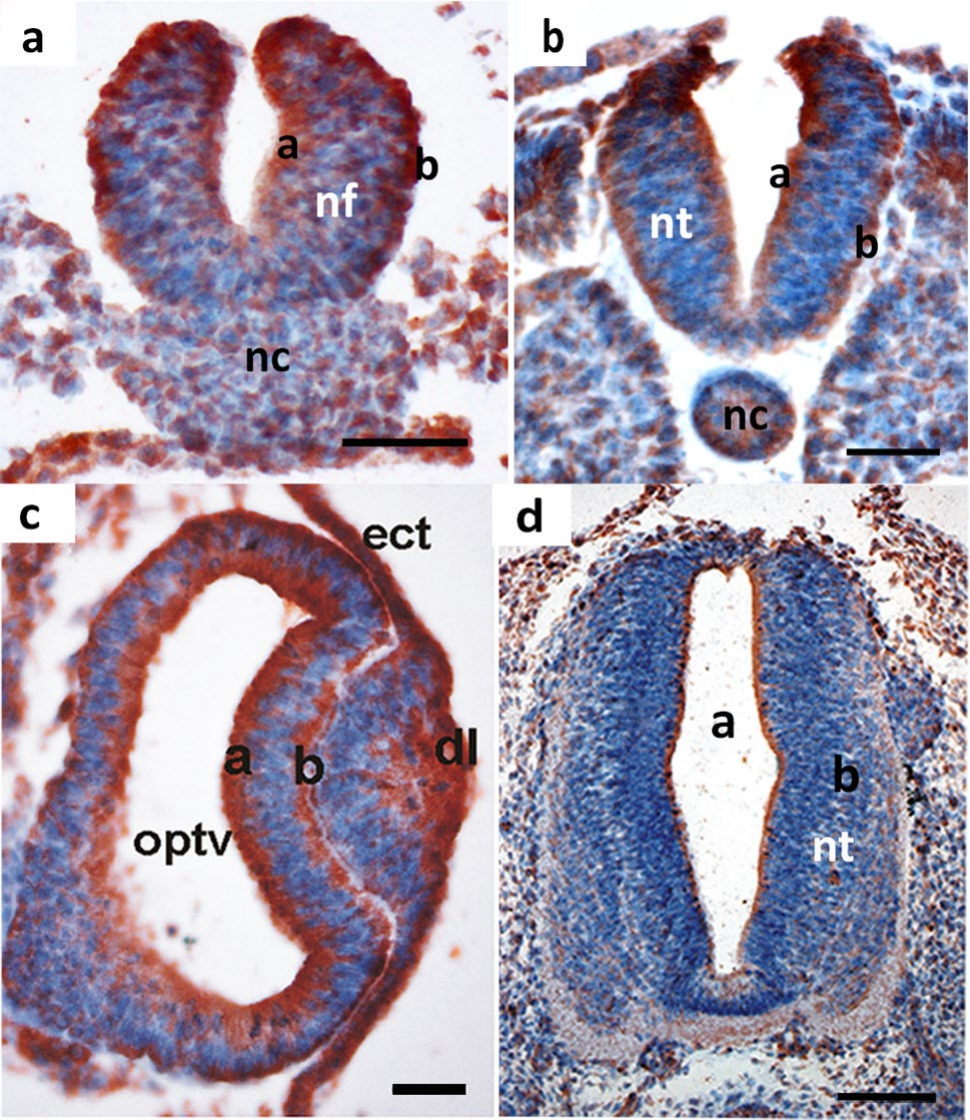
Mdg1/ERdj4 protein pattern in the developing nervous system. **a** HH-stage 12, coronal section. Mdg1/ERdj4 protein accumulates at the apical (a) and basal (b) sites of the neural fold (nf), which has not yet closed to form the roof plate. nc, notochord. Scale bar 50 µm. **b** HH-stage 13, coronal section. Mdg1/ERdj4 protein is present at comparable amounts in the basal (b) and apical (a) zone of the neural tube (nt). Scale bar 50 µm. **c** HH-stage 13, frontal section of the optic vesicle in the developing brain. The polarized Mdg1/ERdj4 distribution is clearly visible in the apical (a) and basal (b) zone of the brain vesicle that forms the optic vesicle (optv). ect, ectoderm; dl, developing lens. Scale bar 50 µm. **d** Chick embryo day 4, the apical zone of the neural tube, which consists of secretorily active ependymal cells, contains high levels of Mdg1/ERdj4 protein. Scale bar 100 µm. Scale bar 100 µm

For the development of the neural tube, the notochord induces the invagination of the overlying epithelial neural plate to form the neural fold with initially unfused neural folds (Massarwa et al. 2014). This epithelial organized neural tissue has increased amounts of Mdg1/ERdj4 protein at the apical and basal margins. The underlying notochord, which is mesenchymally structured, has the mesenchymal-specific Mdg1/ERdj4 pattern as observed in the mesenchymal presomitic mesoderm (Fig. 2a). The ratio of basal to apical protein levels changes during development of the neural tube with initially higher amounts in the basal compartment and later on in the apical compartment: at HH-stage 12, Mdg1/ERdj4 protein staining is most intense at the basal border (Fig. 2a), at HH-stage 13, comparable protein levels are present in the basal and apical region (Fig. 2b). There are always high-protein levels especially at the crest of the neural folds, the region where neural crest cells originate. This strong staining persists until closure of the neural tube.

As being a part of the central nervous system, the optic vesicle exhibits the same polarized distribution of Mdg1/ERdj4 protein as the developing central nervous system with high Mdg1/ERdj4 protein amounts at the basal and apical margins (Fig. 2c). High Mdg1/ERdj4 levels are also present in the epithelium that will differentiate into the optic lens (Fig. 2c). From day 4 (HH-stage 17) onwards, Mdg1/ERdj4 levels are highest in the apical region, where the secretorily active ependymal cells are located, and rather low in the basal zone (Fig. 2d).

### Mdg1/ERdj4 protein pattern in the developing digestive tract

**Fig. 3:**
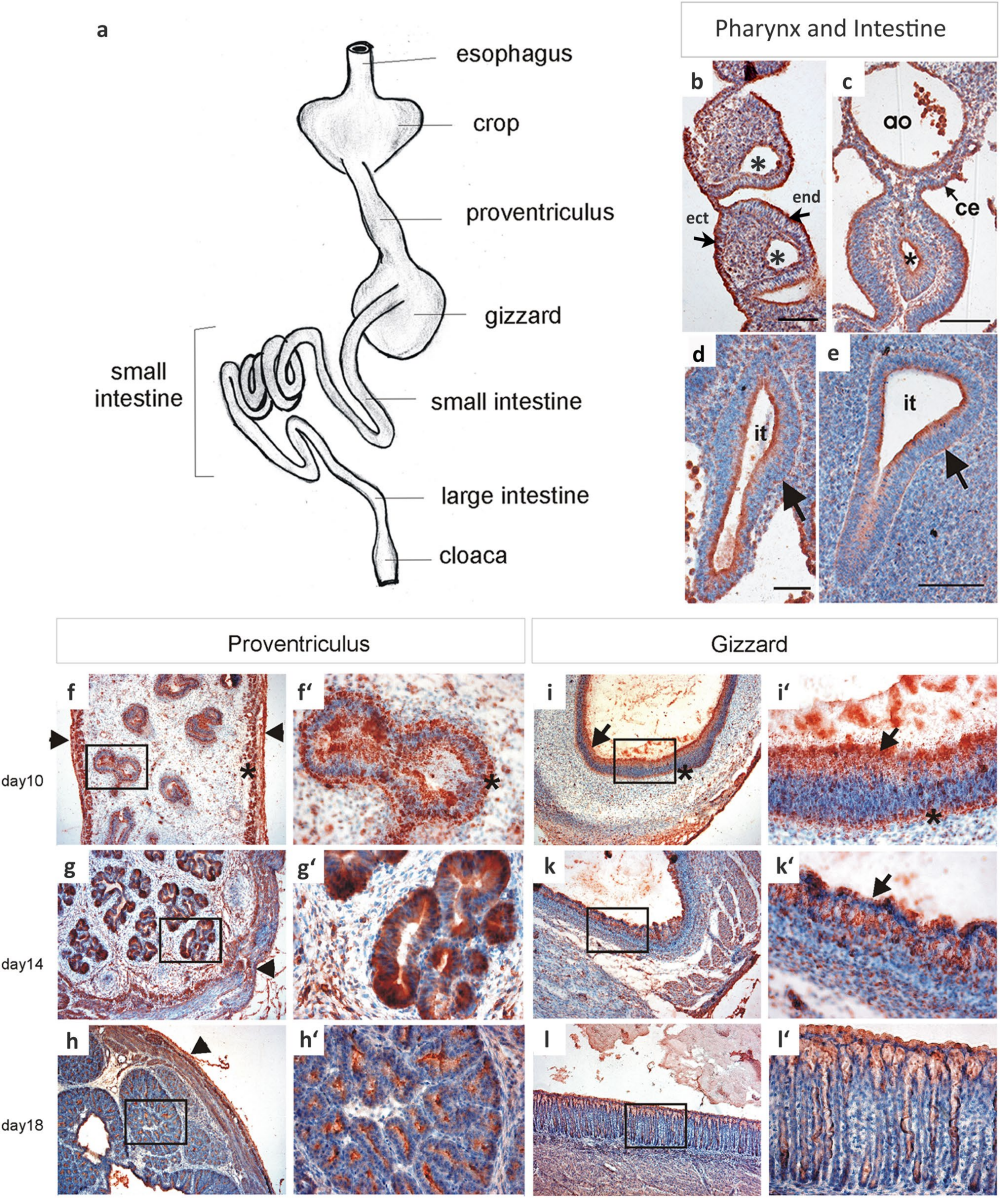
Mdg1/ERdj4 protein pattern in the developing gastrointestinal tract. **a** Schematic drawing of the digestive avian tract with the glandular rich proventriculus located directly before the muscular gizzard. **b** At day 2.5 (HH-stage 17), longitudinal section of the pharyngeal arches. The ectodermal (ect), the endodermal linings (end), and arterial endothelium (*) are Mdg1/ERdj4 positive. Scale bar 100 µm. **c** HH-stage 17, coronal section. The endothelial lining of the aorta (ao), the coelomic epithelium (ce), and the endoderm-derived, apical, epithelial zone of the oesophagus (*) are Mdg1/ERdj4 positive. Scale bar 100 µm. **d** HH-stage 17, intestinal tract in the abdominal region (it). The apical border of the epithelium contains high amounts of Mdg1/ERdj4 protein. In the basal zone, the protein is focussed to a fine-lined border (arrow). Scale bar 50 µm. **e** Embryonic day 4, intestinal tract in the abdominal region (it). The protein pattern observed at HH-stage 17 is maintained (see **d**). Scale bar 50 µm. Immunostaining of the proventriculus (**f**–**h**) and the gizzard (**i**–**l**) at day 10, 14, and 18, respectively. × 10 magnifications (**f**–**l**), corresponding × 40 magnifications of the indicated rectangles are shown in **f′**–**l′**. Arrowheads mark the peritoneum; asterisk marks smooth muscle cells forming layers of the tunica muscularis (**f**) and the lamina muscularis mucosae (**f′**). Arrows point to the luminal epithelium of the gizzard (**i**, **i′**, **k′**). Note the microvilli in **k′** (arrow)

The characteristic epithelial-specific polarized Mdg1/ERdj4 protein pattern also exists in the developing digestive tract. This pattern is found in the pharyngeal arches with high concentrations at the apical pole of the ectodermal and endodermal epithelium. The endothelial linings of the pharyngeal arch vessels and also of the aorta are strongly Mdg1/ERdj4 positive (Fig. 3b, c). The wall of the intestine is positive for Mdg1/ERdj4 with high-protein amounts at the apical pole (at day 2.5, HH-stage 17, Fig. 3c, d). This pattern is maintained until day 4 (Fig. 3e). With maturation of the gut wall, i.e. association of further layers (submucosal and smooth muscle layers) to the endodermal-derived epithelium, Mdg1/ERdj4 protein levels remain elevated but the distribution becomes more uniform throughout the entire epithelium (not shown).

We examined the Mdg1/ERdj4 protein pattern of the organs during further embryonic development by individually dissecting the organs at day 10, 14 and 18. Elevated Mdg1/ERdj4 protein levels were found in the enteric system (proventricle and gizzard). At day 10, Mdg1/ERdj4 protein levels are high in the proventriculus and localized in the apical and basal compartment of the epithelial, glandular cells (Fig. 3f, f′). With further maturation, Mdg1/ERdj4 protein becomes distributed evenly in the entire epithelial cytoplasm (Fig. 3g, g′). At day 18, i.e. shortly before hatching, the cellular Mdg1/ERdj4 protein levels are largely reduced in the glandular epithelium (Fig. 3h, h′). The apical staining is not only within the cells, but also visible extracellularly in the mucus (Fig. 3h, h′). Capillary endothelial cells and the serosa epithelium are Mdg1/ERdj4 positive throughout development.

In the gizzard, Mdg1/ERdj4 protein is localized at the apical and basal border of the lamina mucosa at day 10 (Fig. 3i, i′). Mdg1/ERdj4 protein is not only located within the cells, but also in vesicle-like structures that are secreted luminally (see also Supplementary Fig. 2b, c). With progressive development, Mdg1/ERdj4 protein is high in the microvilli of the epithelial monolayer (day 14; Fig. 3k, k′). At day 18, the Mdg1/ERdj4 signal is evenly distributed at low, "background" levels in all cell layers of the gizzard (Fig. 3l, l′).

We have the impression that the epithelial-specific polarized distribution of Mdg1/ERdj4 protein characterizes the initial process of epithelialization since later on, during further maturation, Mdg1/ERdJ4 protein amounts become evenly distributed throughout the cells and lose their apical/basal localization.

## Discussion

Immunohistochemical stainings revealed that Mdg1/ERdj4 protein is predominantly present in epithelia at various sites: the enterocytes of the gastrointestinal tract, the ectoderm, endoderm, coelomic epithelium, neuroectoderm and vascular endothelium. In general, we observe a polarized distribution in the developing epithelial tissues. Epithelial apicobasal cell polarity is lost when cells are reprogrammed and mesenchymalize (EMT) [see also review (Pei et al. 2019)]. According to the polarized distribution, we hypothesize that Mdg1/ERdj4 is involved in folding and secretion/insertion of membrane proteins such as cell–cell or cell–matrix receptors to organize and maintain the epithelial structure. This is in accordance with its suggested function during terminal differentiation of endothelial cells, i.e. when tube formation is initiated in endothelial cells (Prols et al. 2001; Liu et al. 2014). High Mdg1/ERdj4 protein levels would thus enable the cells to induce and maintain their differentiation state. This hypothesis is substantiated by the evidence that high Mdg1/ERdj4 protein levels lower the metastatic potential of tumor cells (Isachenko et al. 2006). The involvement of Mdg1/ERdj4 in MET has not been directly addressed before. Yet evidence exists pointing to a crosstalk between chaperone signalling pathways and EMT. Overexpression of proteinlysine–tyrosinquinone-dependent copper amino oxidase 2 (LOXL2), a protein known to induce EMT, also activates the IRE1/Xbp1s branch of the unfolded protein response (UPR). Different target genes of Xbp1s have been identified, among them the transcription factors SNAI1 or SNAI2. The expression of SNAI1 and 2 downregulate the cell surface E-cadherin thereby initiating mesenchymalization of the cells (Cuevas et al. 2017). Interestingly, Mdg1/ERdj4 is also a target gene of Xbp1s (Lee et al. 2003; Kanemoto et al. 2005) yet possibly counter acting the EMT process by forming a complex with IRE1/BiP, which leads to monomerization of IRE1 and subsequently the termination of the IRE1–Xbp1s–SNAI1/2-dependent signalling (Amin-Wetzel et al. 2017; Cuevas et al. 2017). Accordingly, inhibition of the IRE-dependent mesenchymalization is mandatory for the initiation of MET.

Controversial data exist with respect to the subcellular localization of Mdg1/ERdj4. In the developing epithelium, Mdg1/ERdj4 protein largely accumulates intracellularly at the apical and basal epithelial borders of the tissue. This is a very interesting finding since the subcellular domain in differentiating epithelial tissue differs from the classical localization, i.e. the endoplasmic reticulum (Shen et al. 2002; Kurisu et al. 2003; Awe et al. 2008; Lai et al. 2012). Growing evidence points to the existence of Mdg1/ERdj4 in several compartments such as the ER, the cytosol (Dong et al. 2008; Lee et al. 2015; Nasr et al. 2018), the extracellular space (Nasr et al. 2019) and even the nucleus (Prols et al. 2001; Berger et al. 2003). The existence of a cytosolic pool has been shown in in vitro experiments (Dong et al. 2008; Lee et al. 2015) and by immunohistochemical stainings of various healthy human tissue types (Nasr et al. 2018). The immunohistochemical stainings shown in this work further reveal extracellular deposition of Mdg1/ERdj4 protein. Extracellular localization of Mdg1/ERdj4 has also been reported as e.g. patients suffering from fibrillary glomerulonephritis exhibit elevated ERdj4 serum levels (Nasr et al. 2019). Moreover, ERdj4 was identified in extracellular glomerular deposits (Andeen et al. 2018; Dasari et al. 2018; Nasr et al. 2018, 2019). The different subcellular pools might point to different compartment-specific functions of Mdg1/ERdj4.

At later developmental stages, high Mdg1/ERdj4 protein levels are preserved in secretorily active cells, as in the enterocytes and ependyme. Elevated levels in secretorily active tissue in the adult have been reported before (Prols et al. 2001; Shen et al. 2002) and most recently in the intestine of adult mice (Huang et al. 2019), but also in nasal epithelial cells, especially in patients with severe cystic fibrosis (Huang et al. 2019). Mdg1/ERdj4-deficient mice suffered from hypoglycemia, high glucagon and reduced glycogen production with reduced beta cell levels while alpha cell levels increased (Fritz et al. 2014). These data further substantiate a central role of Mdg1/ERdj4 in secretion and maturation processes, a hypothesis that has been proposed before (Prols et al. 2001; Shen et al. 2002; Hoshino et al. 2007; Dong et al. 2008) and is now corroborated by the immunohistochemical data shown in this study.

According to the data presented here, we conclude that elevated levels of Mdg1/ERdj4 are essential for the induction of MET and the maintenance of the epithelial organization during embryonic development while constitutive elevated levels are closely linked to secretory activity.

## Electronic supplementary material

Below is the link to the electronic supplementary material.

**Supplementary Figure 1: Immunohistochemical control stainings in the absence of primary antibody. **Immunohistochemical staining of the intestinal tract (it) at day 4 (a, a’), of gizzard at day 6 (b, b’) and day 14 (c, c’) and the proventriculus at day 14 (d, d’) in the presence (left row, a-d) or absence (right row, a’ – d’) of the primary antibody Mdg1/ERdj4. No staining was observed in the absence of primary antibody (TIF 28317 kb)

**Supplementary Figure 2: Mdg1/ERdj4 protein in the developing gizzard. **Maturation of the gizzard epithelium from day 6 to day 18 (day 6 (a), day 8 (b), day 10 (c), day 12 (d), day 16 (e) and day 18 (f)). The basal zone (*) is sharply delineated from day 6 to day 12 (a - d). At the luminal, apical zone small, Mdg1/ERdj4 positive vesicles are seen (b, c). At day 12 the apical enterocytes are strongly Mdg1/ERdj4 positive (d) and during further development the basal zone loses its sharp Mdg1/ERdj4 lineage and Mdg1/ERdj4 protein is primarily present at the apical pole (e, f). Magnification 40x (TIF 26494 kb)
