# Supplementary figures and images for: Protein expression pattern of the molecular chaperone Mdg1/ERdj4 during embryonic development
Source: Histochem Cell Biol. 2020 May 7;154(3):255–63. doi: 10.1007/s00418-020-01881-x (PMC7502036; doi:10.1007/s00418-020-01881-x)

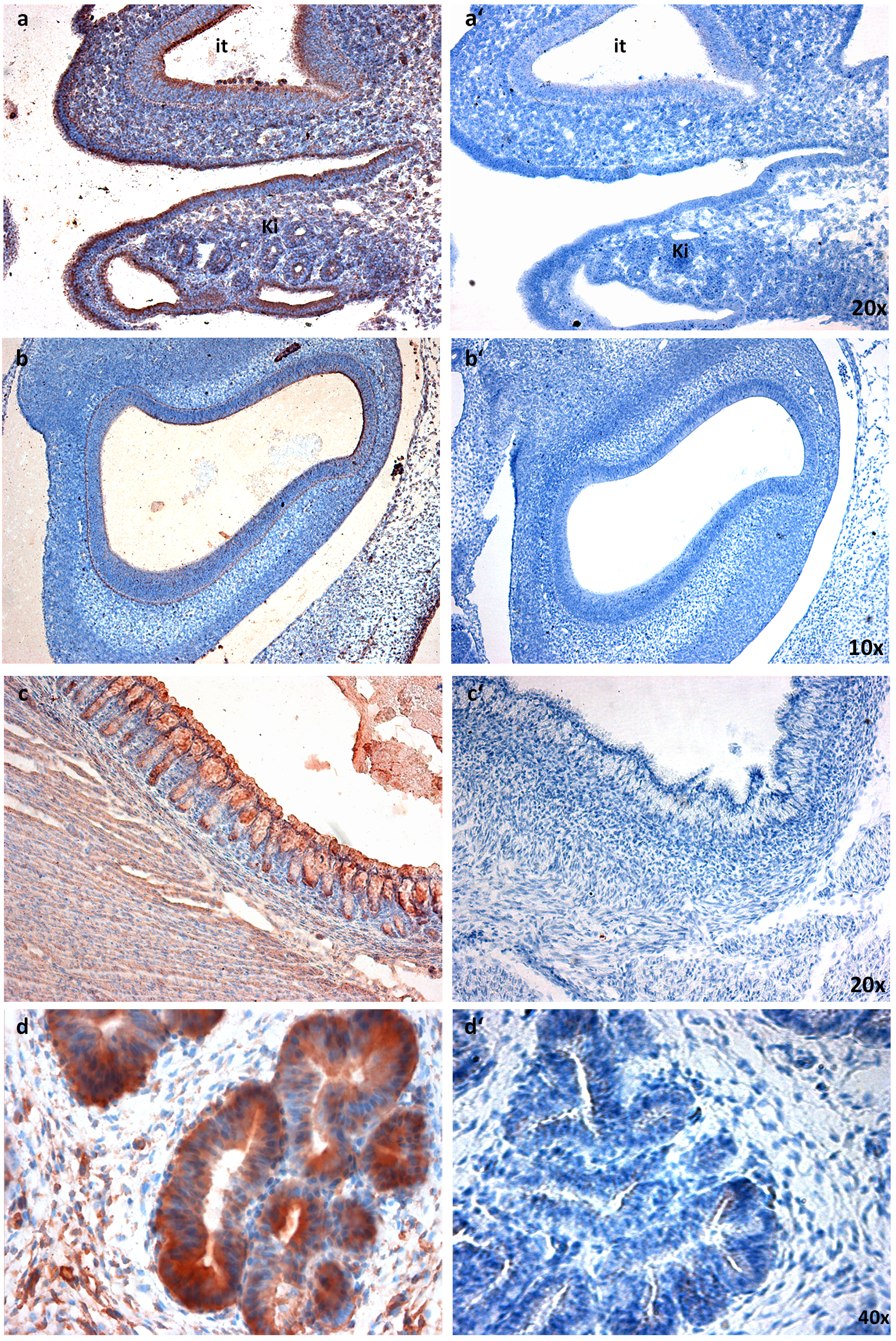

Supplement: Supplementary file 1 — Supplementary Figure 1: Immunohistochemical control stainings in the absence of primary antibody. Immunohistochemical staining of the intestinal tract (it) at day 4 (a, a’), of gizzard at day 6 (b, b’) and day 14 (c, c’) and the proventriculus at day 14 (d, d’) in the presence (left row, a-d) or absence (right row, a’ – d’) of the primary antibody Mdg1/ERdj4. No staining was observed in the absence of primary antibody (TIF 28317 kb) [file 418_2020_1881_MOESM1_ESM.tif]

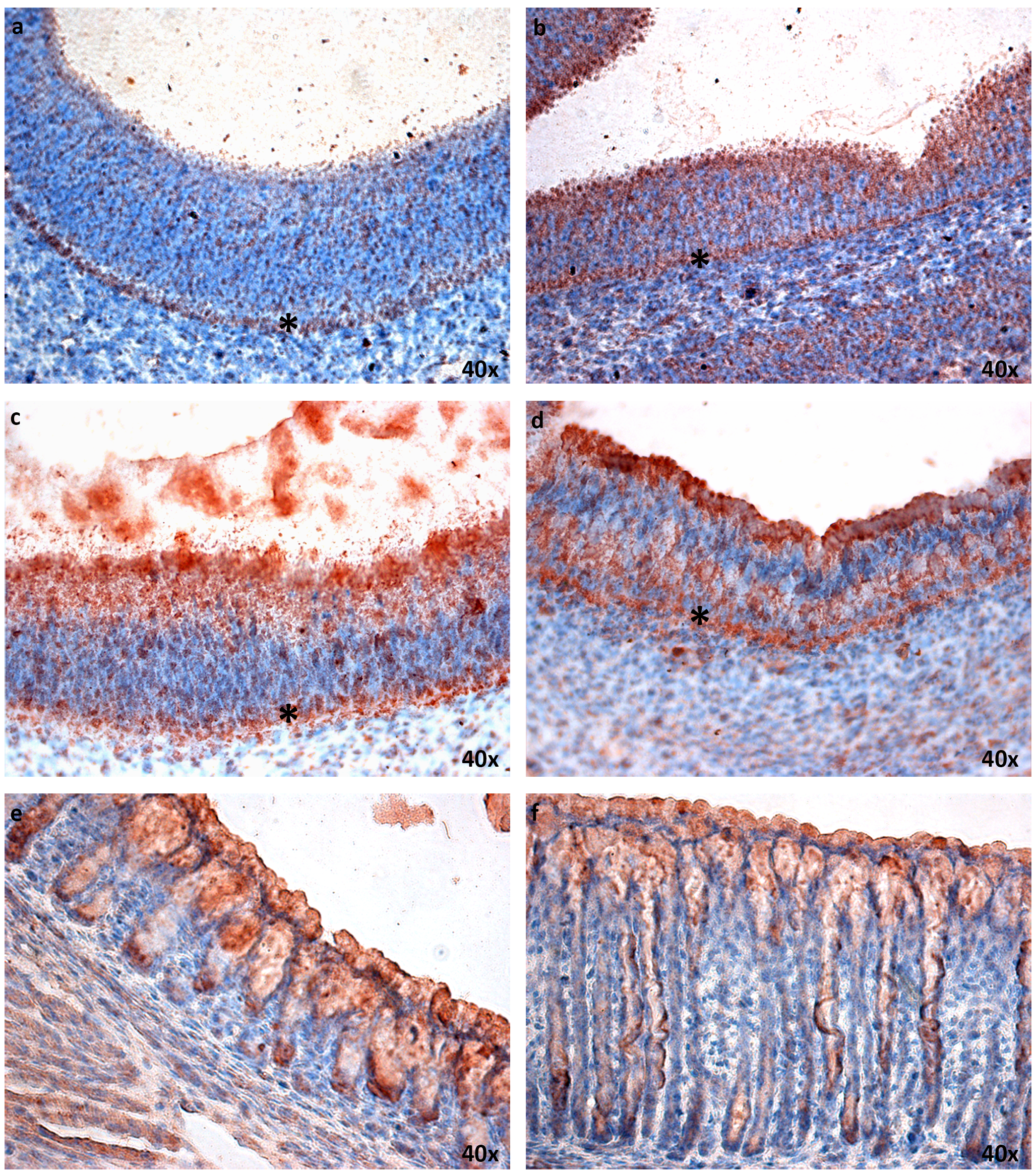

Supplement: Supplementary file 2 — Supplementary Figure 2: Mdg1/ERdj4 protein in the developing gizzard. Maturation of the gizzard epithelium from day 6 to day 18 (day 6 (a), day 8 (b), day 10 (c), day 12 (d), day 16 (e) and day 18 (f)). The basal zone (*) is sharply delineated from day 6 to day 12 (a - d). At the luminal, apical zone small, Mdg1/ERdj4 positive vesicles are seen (b, c). At day 12 the apical enterocytes are strongly Mdg1/ERdj4 positive (d) and during further development the basal zone loses its sharp Mdg1/ERdj4 lineage and Mdg1/ERdj4 protein is primarily present at the apical pole (e, f). Magnification 40x (TIF 26494 kb) [file 418_2020_1881_MOESM2_ESM.tif]
